# Pleiotropy alleviates the fitness costs associated with resource allocation trade-offs in immune signalling networks

**DOI:** 10.1098/rspb.2024.0446

**Published:** 2024-06-05

**Authors:** Reese A. Martin, Ann T. Tate

**Affiliations:** ^1^ Department of Biological Sciences, Vanderbilt University, Nashville, TN 37235, USA; ^2^ Evolutionary Studies Initiative, Vanderbilt University, Nashville, TN 37235, USA

**Keywords:** network robustness, life history trade-offs, coevolution, host–parasite interactions, evolutionary immunology

## Abstract

Many genes and signalling pathways within plant and animal taxa drive the expression of multiple organismal traits. This form of genetic pleiotropy instigates trade-offs among life-history traits if a mutation in the pleiotropic gene improves the fitness contribution of one trait at the expense of another. Whether or not pleiotropy gives rise to conflict among traits, however, likely depends on the resource costs and timing of trait deployment during organismal development. To investigate factors that could influence the evolutionary maintenance of pleiotropy in gene networks, we developed an agent-based model of co-evolution between parasites and hosts. Hosts comprise signalling networks that must faithfully complete a developmental programme while also defending against parasites, and trait signalling networks could be independent or share a pleiotropic component as they evolved to improve host fitness. We found that hosts with independent developmental and immune networks were significantly more fit than hosts with pleiotropic networks when traits were deployed asynchronously during development. When host genotypes directly competed against each other, however, pleiotropic hosts were victorious regardless of trait synchrony because the pleiotropic networks were more robust to parasite manipulation, potentially explaining the abundance of pleiotropy in immune systems despite its contribution to life history trade-offs.

## 1. Introduction

Pleiotropy, where one gene, pathway, or module contributes to multiple discrete life-history traits, is a common feature across the tree of life, with examples found in plants [1,2], animals [3], prokaryotes [4,5] and even viruses [6]. Genetic pleiotropy can be beneficial to organismal fitness by reducing genome size [7], providing architecture for the evolution of novel traits [8,9], or by promoting the evolution of highly fit traits [10]. However, pleiotropy is also associated with life-history trade-offs stemming from evolutionary constraints on protein function [10–12], resource allocation conflicts [13] or the deployment of the same genes to express traits that may not be correlated [3].

These conflicts take on outsized importance when considering the evolution of immune systems. In taxa as disparate as plants, insects and humans, immune genes play double duty in defending against parasites and driving other important traits like development; immunity is even among the most overrepresented processes in studies of pleiotropy in humans [14]. This posits a fundamental conflict of interest, since immune systems tend to evolve rapidly to keep up with parasitic antagonists while developmental pathways tend to be under purifying selection to prevent catastrophic effects on organismal fitness [15,16]. In the fruit fly genome, for example, pleiotropic immune genes are significantly more likely to be under purifying selection than genes not involved in developmental processes, particularly if they are expressed early in development or broadly across life stages [17]. This raises the question: if pleiotropy constrains the adaptive evolution of immune genes, why is it so common?

It is possible that compensatory evolution can erase some of the evolutionary conflict that stems from using a single gene in multiple traits [18], but this does not resolve the issue of deployment. After all, if the same tissue needs to mobilize the same intracellular signalling pathway in the same life stage to control two traits that differ in the optimal magnitude of their induction, or if a limiting resource or protein prevents optimal investment in two traits simultaneously, then a trade-off is inevitable (table 1). For example, the synchronous deployment of the phenoloxidase pathway for immunity and development drives trade-offs in recently molted *Lymantria dispar* larvae [26], as the melanin precursors that would normally be used to encapsulate bacteria have been diverted to cuticle tanning. Another possibility is that pleiotropy provides a transient benefit during the evolution of new traits by lending well-functioning modules to novel traits [8]. In such a case, however, pleiotropy should only be present in young networks or new traits, not tightly conserved across broad swaths of evolutionary time (e.g. the Toll pathway and other examples in table 1). A final possibility is that there is some benefit to pleiotropy at higher levels of biological organization that is not apparent when considering constraints on an individual gene or module. A potential benefit could arise from honed control of variability over the expression of pleiotropic proteins [28], imparting a kind of robustness to the rest of the signalling network. Such robustness is vital to host fitness in the face of immune challenge [29,30], in part because parasite disruption of host immunity is extremely common and networks that lack robustness are particularly vulnerable to manipulation [15].

**Table 1.** Biological examples of pleiotropic resource allocation trade-offs.

gene/pathway	timing of trade-off	traits	system	parasite	summary	references
InR	asynch.	*growth/fecundity* and *lifespan/stress tolerance*	*D. melanogaster*	N/A	the insulin-like receptor (InR) in *D. melanogaster* is associated with trade-offs between fecundity and development time versus lifespan and stress tolerance	[19]
RXFP2	asynch.	*fecundity* and *organismal survival*	*Ovis aries*	N/A	relaxin-like receptor 2 (RXFP2) is associated with a trade-off between reproductive success and year over year survival	[20]
RPM1	asynch.	*infection resistance* and *seed production*	*A. thaliana*	*P. syringae*	the *RPM1* gene confers resistance to *Pseudomonas syringae* in *A. thaliana* but reduces reproductive fitness	[21,22]
TNFa	asynch.	*immune organ development* and *offspring quality quantity ratio*	TNF-KO mouse	N/A	TNFa is critical for immune homeostasis and maintains the quantity versus quality trade-off in mouse litters	[23]
(HIF)-1*α*	synch.	*immune defence* and *wound healing*	(HIF)-1*α* KO *Mus musculus*	group A *streptococcus* strain 5448	hypoxia-inducible transcription factor (HIF)-1*α* allows NK cells to mediate a metabolic trade-off between wound healing behaviour and bacterial defence	[24]
juvenile hormone (JH) and 20 hydroxy ecdysone (20E)	synch.	*AMP production* and *egg production*	review of insects	N/A	JH is an antagonist of immune function while 20E is a potentiator of immunity. The balance between JH and 20E is vital for egg maturation	[25]
ProPO	synch.	*pathogen melanization* and *cuticle tanning*	*Lymantria dispar*	*LDMNPV*	phenoloxidase is used to both tan insect cuticles and melanize pathogens, this leads to lowered resistance to infection immediately following moulting	[26]
Toll	synch.	*AMP production* and *growth*	Gal4/UAS *D. melanogaster* constitutively expressing Toll^10b^	N/A	suppression of insulin signalling following the activation of Toll signalling in the fat body of *Drosophila melanogaster* suppresses allocation of resources to growth	[27]

We hypothesized that both resource scarcity and synchronous deployment of two traits should exacerbate trade-offs among traits, and by extension the fitness costs of pleiotropy, thereby promoting the decoupling of pleiotropic signalling pathway architecture into two discrete traits. When the deployment of traits occurs asynchronously, on the other hand, or resources are plentiful, the costs of simultaneous deployment should be diminished. If these costs are sufficiently diminished, then the benefits of maintaining fewer genomic modules could promote the maintenance of pleiotropy [7]. We also considered that since pleiotropy appears to be particularly common in immune systems, something about the evolution or coevolution with parasites, i.e. feedback from different levels of biological organization, could provide benefits to the maintenance of pleiotropic architecture.

To address this hypothesis, we developed a theoretical model of signalling network co-evolution featuring host and parasite populations. Drawing inspiration from the partially pleiotropic Toll and ProPO pathways in insects (table 1), hosts are defined by a pair of signalling networks, one developmental and the other immune. Signalling networks that control each trait can be fully independent (independent effector hosts) or feature two partially decoupled signalling networks that share a single effector or downstream module (shared effector hosts). Host life history includes an immature stage where they are subjected to developmental signals and an adult stage where there is no developmental signalling. Parasites are defined by a single network that allows them to interfere with host immune signalling, a common form of host–parasite interaction that is thought drive host immune signalling network evolution [15]. In our model, hosts defend against parasitic infection during their developmental period or following the end of development, creating a synchronous or asynchronous signalling environment. Hosts evolve in one of three resource conditions (scarce, plentiful, alternating) which limit the actions of proteins in their signalling networks. We use this modelling framework to investigate the conditions that favour the evolutionary maintenance of pleiotropy despite the proximate and ultimate constraints it imposes on adaptation, revealing benefits to pleiotropy that manifest in heterogeneous populations.

## 2. Methods

We adapted a framework originally designed to model the evolution of generic signalling networks [31] and subsequently modified to study immune signalling network robustness [32], the evolvability of immune responses during co-evolution [29] and the interaction between pleiotropy and immune response dynamics during co-evolution [10]. Here we have revised the model to include two signalling networks that can function independently but have a shared resource pool. While these modifications were originally made to reflect pleiotropy between immune and developmental processes, the model itself is generalizable to any set of pleiotropic traits. Aside from sharing effector modules (downstream pleiotropy), we also allowed hosts to develop inter-network connections (upstream pleiotropy) to study the fitness effects of pleiotropic interactions between two networks as well as the conditions that promoted the creation and maintenance of such connections.

For the purposes of this study, we broadly defined an effector as a module of genes (e.g. the intracellular portion of the Toll pathway), a regulatory element or output (e.g. melanin from phenoloxidase). Hosts with a shared (pleiotropic) effector have a single downstream module controlled by both signalling networks, while this is decoupled in hosts with independent effectors. In all simulations, each host was initially composed of two networks, with independent effector hosts having a detector, three signalling proteins and an effector in each network, and shared effector hosts having a detector and three signalling proteins in each network, but a single effector shared by both networks.

Connections between proteins and the regulatory behaviour of those connections were randomized during the initiation of a simulation. Host creation parameters were derived from previous agent based models of signalling network evolution [10,29,31,32]. Host mutations occurred during reproduction and resulted in the duplication or deletion of signalling proteins, or the addition, deletion, or modification of regulatory interactions between proteins in the network. The only restrictions to this evolutionary process were that detectors and effectors were not allowed to develop a direct connection and could not be deleted. Hosts were required to maintain at least one signalling protein, preventing them from evolving an entirely non-functional immune network. Host fitness was expressed as a function of immune effector abundance, cumulative parasite abundance, and how closely they matched a developmental input (see §2a(vi)). Parasite fitness was derived from cumulative parasite abundance, a proxy for transmission potential.

To evaluate the evolutionary implications of hosts using a shared effector rather than multiple independent effectors we designed two major classes of simulation: co-evolutionary, designed to establish a fitness landscape to identify resource and signalling conditions that favored independent or shared effector hosts, and competitive simulations, designed to evaluate the competitive fitness of shared and independent effector hosts against each other. During co-evolutionary simulations, a population of hosts (*n* = 500) evolved for 1000 generations with a population of parasites (*n* = 250). The initial hosts and parasites in a population can be thought of as founder ‘genomes’, seeding the simulation with initial genetic variance for evolutionary processes to act on. Each host had a 50% chance of being infected by a parasite in each generation.

Competitive simulations consisted of two phases: independent evolution and competition. During the independent evolution phase of a competitive simulation a population of hosts with independent effectors (*n* = 250) and a population of hosts with shared effectors (*n* = 250) were allowed to co-evolve with parasite populations (*n* = 125) in isolation from the other host population. As in the independent evolution case, the initial hosts and parasites can be thought of as being a founding ‘genotype’ from which a successful lineage may descend. After 250, 500, or 1000 generations of independent evolution, the simulation entered the competition stage, combining the host populations along with their parasites. The competition ended when one population died out entirely or 1000 generations had passed with no winner (draw). Host and parasite population sizes and the number of generations were chosen to achieve representative and repeatable dynamics while conserving computational resources [33]. These simulations allowed us to evaluate the competitive costs and benefits associated with sharing an effector between multiple traits.

### (a) Simulations of independent evolution

Evolutionary simulations were carried out in the Julia programming language (v 1.6) [34]. Each simulation is composed of generations which can be broken down into the following discrete events. The initialization step happened only once per simulation.

#### (i) Initialization

A population of 500 shared effector or independent effector hosts is generated. All independent effector hosts start with a detector, three signalling proteins, and an effector for both their immune and their developmental signalling networks. All shared effector hosts start with a detector and three signalling proteins for their immune and developmental signalling networks but share a single effector. Connections are established between proteins in a signalling network at random, with each valid connection in the network having a 50% chance of occurring. Each connection is assigned a randomly selected regulatory value from the interval [−1,1]. A population of 250 parasites is also generated at the start of each simulation. Parasite signalling networks are simple compared to the host signalling network, having only a target signalling protein, regulatory behaviour on that target, and a self-targeted up-regulatory action to represent within-host reproduction.

#### (ii) Host equilibrium

Each protein *p* in an immune network has a total concentration equal to 1, with the active portion denoted P_i_* and inactive portion denoted *P_i_* ([P_i_*] + [*P_i_*] = 1). When determining the effects of protein *P* on other proteins in the network only the active portion is considered. During infection, changes in parasite abundance are calculated as though it was another protein in the network. The change in $P_{i}^{*}$ is determined by the regulatory action on *P_i_* defined as

2.1 $$\frac{d[P_{i}^{*}]}{dt}=\left(\left[P_{i}]\underset{j}{\sum }k_{i,j}[P_{j}^{*}\right]\right)-\left(\left[P_{i}^{*}]\underset{j}{\sum }I_{i,j}[P_{j}^{*}\right]\right)-\mathrm{Protein}\;\mathrm{Use},$$

2.2 $$\mathrm{Protein}\;\mathrm{Use}\;=\;\mathrm{UseCoef}*(\mathrm{Num}(k_{i})+\mathrm{Num}(I_{i})),$$

where $k_{i,j}$ are the upregulatory coefficients from protein *P_j_* to protein *P_i_* and $I_{i,j}$ are the downregulatory coefficients from protein *P_j_* to protein *P_i_*. Here upregulatory connections are those that increase the abundance of the target protein and downregulatory connections are those that decrease target protein abundance. The term *Protein Use* describes the inactivation of some $P_{i}^{*}$ owing to the action of *P_i_* on other proteins in the network, for all simulations UseCoef= 0.01 so for each interaction in a timestep a protein *P_i_*'s total active portion, [P_i_*], decreased by 0.01* (the number of interactions from *P_i_*). A larger UseCoef would necessitate fewer interacting partners or a greater upregulation of protein *P_i_* to maintain [P_i_*].

Starting from initial concentrations of P* = 0.5 for all proteins in the network, P* for each protein at time step *t + 1* is calculated numerically using equation (2.1) for five time-steps to allow host signalling to reach equilibrium.

#### (iii) Development

Following the equilibrium period, developmental signalling begins, defined by the following equation:

2.3 $$\mathrm{Dev}.\;\mathrm{Signal}=\sin\left(\frac{x(t)}{2}\right)+0.5,$$

where *x*(*t*) are the sequential values in range(0,8π,50) at time-step *t*. Developmental signalling occurred for 50 time-steps in all simulations. This signalling dynamic was chosen to mimic a pulsed or periodic developmental signal used to coordinate periodic events like periodic segmentation, a process common in both vertebrates and invertebrates [35].

#### (iv) Infection

In each generation, each parasite infected a single host, resulting in an infection prevalence of 50%. Hosts were infected at random. Mechanistically, the infecting parasite was treated as a new protein in the host immune signalling network. The asynchronous simulation results in the main text reflect the scenario where infection occurred in a 50 timestep window following the end of the developmental period. For symmetry we also conducted a subset of simulations where developmental signalling took place following infection; see §2a(v). In synchronous simulations, hosts were infected at a randomly selected point during the developmental period. Parasites are treated as an additional protein in the host immune network with an upregulatory connection of 1 to the host detector, a self-targeted upregulatory connection of 0.8 to represent replication, and an up- or downregulatory connection to a signalling protein targeted by the parasite. The parasite reproduction rate of 0.8 was chosen to align with previous agent based models of host parasite co-evolution [10,29]. The immune effector (or sole effector in the case of the shared effector hosts) of the host network gains a downregulatory connection of −1 to the parasite.

#### (v) Host life

Equation (2.1) was used to calculate changes in protein and parasite abundance for 150 time-steps, corresponding to the full host lifespan. For the first 50 time-steps following the equilibrium period the hosts received developmental input, and synchronous hosts were infected. For the proceeding 100 time-steps there was no developmental input, and the asynchronous hosts were infected. We also conducted a smaller number of simulations where the two periods were reversed, so that the 100 time-steps of no developmental input occurred prior to the 50 time-steps of developmental signalling, flipping the infection timing for the asynchronous and synchronous simulations. These results are reported in the electronic supplementary material.

#### (vi) Fitness calculation

Using data from the host life phase, host fitness was calculated using the following equation:

2.4 $$W_{\mathrm{Host}}=\;e^{-(\mathrm{Imm}.\;\;\mathrm{Eff}.\;\;\mathrm{Area}\;+\;\mathrm{Par}.\;\;\mathrm{Area}\;+\;\mathrm{Dev}.\;\;\mathrm{Cost})}\;.$$

With

2.5 Dev. Cost=(1−Corr(Dev. Sig.,Dev. Out.))+mean(abs(Dev. Sig.−Dev. Out.).

Imm. Eff. Area refers to the normalized area under the curve of immune effector abundance. Immune effector area is normalized to the total length of the hosts life. Par. Area refers to the normalized area under the curve of parasite abundance. Parasite area is normalized to the length of time from infection start to the end of host life. Dev. Cost is our metric for capturing how closely hosts follow the developmental signal described in equation (2.3) with Dev. Sig. being the input developmental signal and Dev. Out. being the effector output. This captures both the mean absolute difference between host developmental effector abundance and developmental signal and the correlation between host developmental effector abundance and developmental signal. Parasite fitness was derived from the normalized area of the parasite infection, akin to the total number of parasites produced over the course of the infection.

#### (vii) Host and parasite death

In each generation up to 30% of each population died, with hosts that succumbed to infection taking priority in the death process, following previously published standards [10]. Host deaths were first determined based on those that had failed to appropriately control their infection, meaning that Par. Area (defined in §2a(vi)) exceeded the death threshold of 0.9 [10]. If less than 30% of the population died due to parasite burden, surviving individuals were selected to die in a fitness-weighted manner. At random an individual was selected from the population and its chance of dying was inversely proportional to its relative fitness against the population. This process continued either until all surviving hosts had been evaluated once, or until 30% of the population had died. In the case where greater than 30% of either population died due to poor fitness or uncontrolled infection, the excess deaths were added back to the healthy population following random selection. Population size and deaths were capped as a concession to the low fitness of initial randomly generated networks and computational expenses. Parasite deaths were determined strictly based on fitness, with the 30% of the parasite population that was least fit being selected for death in each generation.

#### (viii) Host reproduction

Survivors were picked in a fitness-weighted manner to reproduce, with a host's chance to reproduce being proportional to its relative fitness in the population. The population in the proceeding generation was composed of all hosts that survived the death process, and as many offspring from the surviving hosts as was necessary to bring the host population back to 500 or 1000 individuals for independent and competitive simulations respectively. Reproduction resulted in the creation of a direct copy of the parent or, rarely, a mutated copy (mutation rate: 5 × 10^−3^). For hosts, a mutation could be one of the following modifications to the parent network: (1) add a protein–protein interaction between two randomly selected proteins with a random regulatory behaviour (relative probability = 0.25), (2) delete a protein–protein interaction (relative probability = 0.25), (3) alter regulatory coefficient (relative probability = 0.3), (4) delete a protein (relative probability = 0.1), (5) duplicate a protein (relative probability = 0.1).

#### (ix) Parasite reproduction

To reflect the expectation that parasites that achieve a higher within-host abundance would pass their infection on to more hosts than those that had a lower abundance, the number of offspring a parasite created at the end of a generation depended on their within-host abundance. Parasites with an abundance between 0 and 0.33 produced one offspring, those with an abundance between 0.34 and 0.66 produced two offspring, and an abundance greater than 0.66 generated 3 offspring. Parasites reproduced until the population was completely replenished and reproduced in order of their fitness in the population, from most fit to least fit. Parasite offspring directly copied the parent, or rarely a mutated copy was produced (mutation rate: 1 × 10^−2^). Parasite mutations are defined as follows: (1) randomly change the target signalling protein (relative probability = 0.5), (2) change the regulatory behaviour on the targeted signalling protein to a new value in [−1,1] (relative probability = 0.5).

### (b) Competitive simulations

Competitive simulations began with a burn-in period of isolated evolution, as described above, for 250, 500, or 1000 generations. During the isolated evolution phase of the simulations, populations of hosts with shared effectors (*n* = 250) and hosts with distinct effectors (*n* = 250) co-evolved with their own parasites (*n* = 125). When the isolated evolution period ended, the shared effector and distinct effector host populations were combined along with their respective parasite populations. Hosts competed in a fitness-weighted manner for space in the next generation and competition was resolved when either one population of hosts went extinct, or the competition had continued for 1000 generations.

### (c) Resource limits on protein activation

To simulate a restriction on signalling network activity associated with limited resources, we restricted the creation of new active protein per time-step based on resource availability. When resources were scarce, we limited the generation of new active protein to 0.1 meaning $\sum d[P_{i}^{*}]/dt\leq \;0.1$ for all positive changes in $\left[P_{i}^{*}\right]$. When activation of new proteins exceeded 0.1, all upregulatory behaviour was proportionally reduced such that $\sum d[P_{i}^{*}]/dt=0.1$. When resources were plentiful, generation of new active protein was limited to 1, $\sum d[P_{i}^{*}]/dt\leq \;1$. As with the scarce resource condition, when this inequality was violated by regulatory activity, all upregulatory behaviour was proportionally reduced until $\sum d[\;P_{i}^{*}]/dt=\;1$.

### (d) Host robustness

To determine if hosts with shared effectors were more susceptible to parasitic manipulation than non-pleiotropic hosts, we calculated the mean absolute difference in active immune effector abundance between intact hosts and hosts with a single signalling protein knockout. The knockout network was generated by removing a single signalling protein from the network, a standard approach when studying network robustness [29,32]. Hosts were infected with a non-disrupting parasite (a parasite that could not interfere with host signalling proteins) and completed steps (2)–(5) of independent evolution as described above. The mean of the absolute difference between the intact and knockout networks effector abundance was calculated and is used as a metric of the network reliance on a specific signalling protein to produce the evolved response. See electronic supplementary material, figure S1, for a diagram of the host robustness calculation process. Within a resource condition, independence of samples was determined using a Kruskal–Wallis non-parametric ANOVA and if significant differences were detected, multiple comparisons were carried out using pairwise Mann–Whitney *U* tests.

### (e) Network features

All network features were determined for the most common host in a population at the end of independent simulations. Network connectivity was calculated by counting the number of protein–protein interactions in the immune and developmental networks and dividing that number by the number of possible connections those networks could possess. Only connections within networks were considered for network connectivity calculations and the independence of samples was determined using a Kruskal–Wallis non-parametric ANOVA. If significant differences were detected within a resource condition, multiple comparisons were carried out using pairwise Signed Rank tests. Network size was determined by counting the number of proteins present in both the immune and developmental network, the reported network size for independent effector hosts was reduced by one to adjust for the advantage of starting with 10 proteins rather than 9 in the shared effector case. The independence of samples within a resource condition was determined using a Kruskal–Wallis non-parametric ANOVA and *post* *hoc* pairwise Signed Rank tests. Statistical comparisons of variance between samples, such as the variance in absolute fitness values attained by a population, were conducted using the variance *F*-test with significance threshold adjusted by Bonferroni correction.

### (f) Quasi-binomial regression

To determine the probability of shared effector hosts winning a competitive simulation we calculated a binomial regression with the logistic link function using signalling timing, generations prior to competition, and resource availability as predictors of the final proportion of the population composed of shared effector hosts. Because these proportions were not strictly binomial, we adjusted the traditional regression by estimating the dispersion parameter of the quasi-binomial distribution and using this parameter to update the standard errors of the regression. The model fit was proportion of population that shares an effector approximately synchronous signalling + resource availability + generations of evolution prior to competition.

## 3. Results

### (a) The fitness benefits of independent effectors depend on synchrony and resource availability

**Figure 1.**
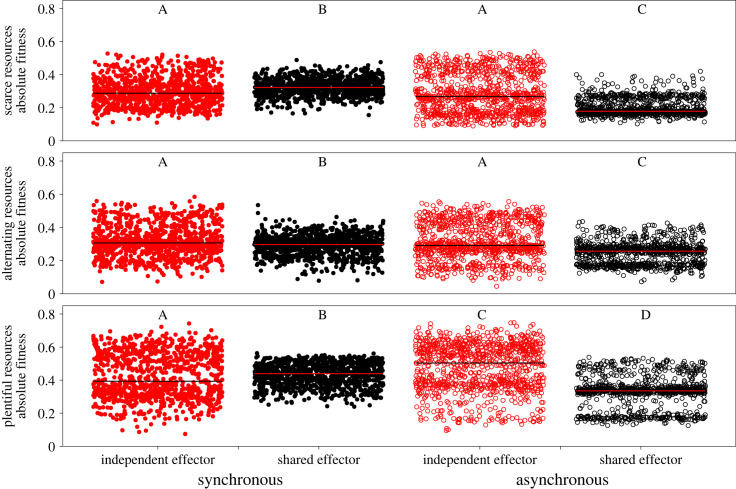
Independent effector hosts tend to be significantly more fit than shared effector hosts. Each dot represents the arithmetic mean fitness of the shared (black) or independent effector population (red) in the final generation of a simulation for synchronous (filled dots) or asynchronous (open dots) signalling conditions. Top row = scarce resources, middle = alternating resources, and bottom = plentiful resources. Within a resource condition, independence of samples was determined using a Kruskal–Wallis non-parametric ANOVA and if significant differences were detected, multiple comparisons were carried out using pairwise Signed Rank tests. Groups that share a letter are not significantly different from each other and letters are re-used between resource conditions.

After the final generation of independent evolution, hosts with independent effectors attained significantly higher absolute fitness than shared effector hosts across all resource conditions when trait expression was asynchronous, as well as under variable resource conditions with synchronous signalling. Shared effector hosts attained significantly higher fitness only when immune and developmental activity were synchronous and resources were scarce (figure 1). Scarce resource environments produced hosts that were less fit than those in plentiful resource environments under the same signalling conditions. Hosts that evolved in the alternating resource condition attained intermediate absolute fitness levels. When looking at simulations where the developmental signalling period occurred in the final 50 time-steps of the simulation, the same broad trends in absolute fitness differences are observed (electronic supplementary material, figure S2), demonstrating that these findings are robust to the order of trait expression.

We observed drastic differences in the variance around the mean of host population fitness, however. In all resource and signalling conditions, hosts with independent effectors had significantly higher absolute fitness variance as determined by the variance *F*-test. The asynchronous signalling condition resulted in universally elevated absolute fitness variance, and within each scenario the plentiful resource condition resulted in greater fitness variance than either the alternating or scarce conditions (electronic supplementary material, table S1). In some signalling and resource conditions, we found what seem to be bi- or even trifurcations in host population fitness across simulations (figure 1); further investigation showed that these bifurcations largely arose from early adaptative events, with hosts being set on a low or high fitness trajectory in the first 100 generations of a simulation. Importantly, a host population was not locked to this early trajectory, with escape from the low fitness trajectory being rare, but occurring throughout the remaining generations of a simulation.

To quantify the effects of the variance in arithmetic mean fitness, we calculated the geometric mean fitness of each population across the first 50 generations of competitive simulations for the scarce resource condition when signalling was synchronous or asynchronous (electronic supplementary material, tables S3 and S4). In all cases, the difference between the arithmetic mean and the geometric mean was greatest in the independent effector population. In most cases the difference between the geometric mean fitness of the shared and independent effector population was smaller than the difference between the arithmetic mean fitness of those populations.

### (b) Shared effector hosts have a competitive advantage over independent effector hosts

**Figure 2.**
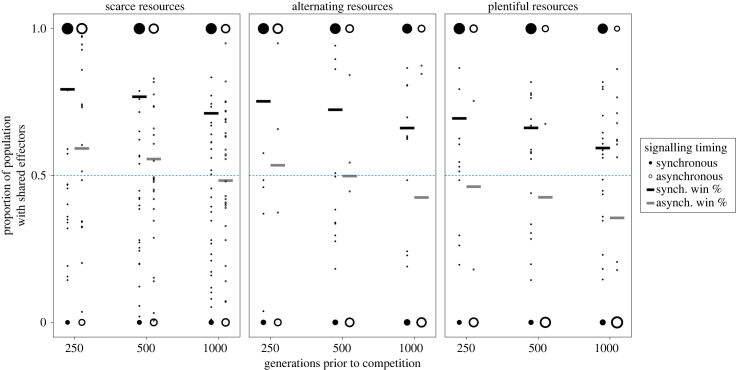
Shared effector hosts outcompete independent effector hosts in most conditions. The black lines show the predicted probability of shared effector hosts winning a competitive simulation when signalling was synchronous, the grey lines show the predicted probability of shared effector hosts winning a competition when signalling was asynchronous. In both cases, the predicted probability was determined by a quasi-binomial regression on the proportion of the population that had a shared effector in the final generation of a simulation. The *y*-axis shows the proportion of the host population that was composed of shared effector hosts, with the size of dots corresponding to the number of simulations that ended with that proportion. The *x*-axis shows the number of generations that were allotted for independent evolution prior to competition beginning.

Despite their lower arithmetic fitness in most scenarios, hosts with shared effectors outcompeted hosts with independent effectors, winning more than 50% of competitive simulations in nearly all signalling and resource conditions. Shared effector hosts enjoyed significantly higher win percentages when signalling was synchronous relative to asynchronous. Independent effector hosts won the majority of competitive simulations when signalling was asynchronous and resources were plentiful or alternating (following 500 or 1000 generations of independent evolution). Across all resource and signalling conditions, independent effector hosts became more competitive when the burn-in period prior to competition was longer (figure 2).

The predicted chance of a shared effector host winning a simulation was determined using quasi-binomial regression and revealed that synchronous signalling (*p* < 0.001), the number of generations of evolution prior to competition (*p* < 0.001), and resource availability (*p* < 0.001) all contributed significantly to shared effector host victory. The greatest coefficient from the regression (electronic supplementary material, table S2) was associated with signalling timing, with shared effector hosts undergoing synchronous signalling having 2.6 times the odds of winning a competitive simulation relative to those undergoing asynchronous signalling.

### (c) Hosts with pleiotropic effectors are robust and highly developmentally fit

**Figure 3.**
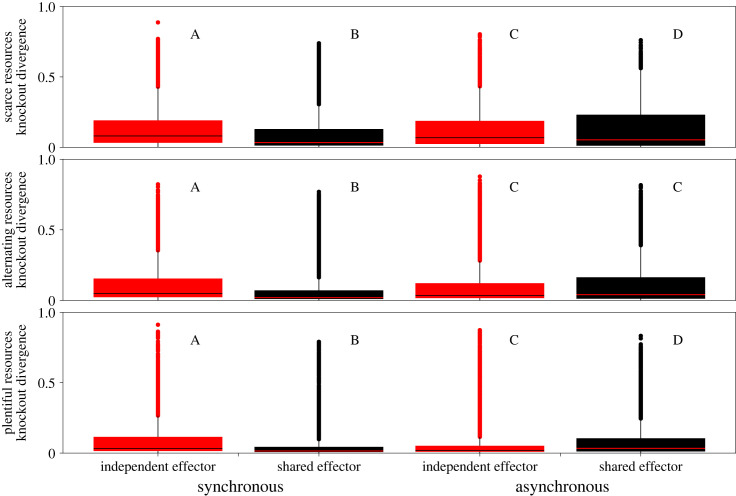
Shared effector host immune responses tend to be more robust than independent effector host immune responses. Host signalling network robustness was measured as the mean absolute difference between immune effector activity in intact and knockout hosts, using the most common hosts from the end of independent evolution simulations as the population of intact hosts. Higher values indicate networks were less robust; see §2d for detailed methods. The *y*-axis shows the mean absolute difference between the knockout immune response and the intact host immune response. The columns indicate the resource availability of the simulations intact hosts evolved in. Black/red lines are mean change in effector abundance following knockout. Within a resource condition, independence of samples was determined using a Kruskal–Wallis non-parametric ANOVA and if significant differences were detected, multiple comparisons were carried out using pairwise Mann–Whitney *U* tests. Groups that share a letter are not significantly different from each other and letters are re-used between resource conditions.

When signalling was synchronous, shared effector hosts were significantly more robust to parasite manipulation than hosts with independent effectors. When signalling was asynchronous, shared effector hosts were significantly more robust than independent effector hosts when resources were scarce, while hosts with independent effectors were significantly more robust than shared effector hosts when resources were plentiful. Universally, hosts in plentiful or alternating resource conditions were more robust than in scarce resource conditions (figure 3). When looking at simulations where the developmental signalling period occurred in the final 50 time-steps of the simulation, the same broad trends in host robustness are observed (electronic supplementary material, figure S3), showing that our findings with regards to host robustness are not sensitive to the order of signalling events.

Hosts with independent effectors paid lower immune costs than hosts with pleiotropic effectors across all signalling and resource conditions. These fitness cost differences were greater when signalling was asynchronous and when resources were scarce (electronic supplementary material, figure S4). The parasite-associated fitness costs paid by shared effector hosts tended to be significantly lower than those of the independent effector hosts when signalling was asynchronous; this relationship was reversed for synchronous situations (electronic supplementary material, figure S5). Across nearly all conditions, shared effector hosts paid significantly lower developmental costs than hosts with independent effectors, and this difference was greatest when signalling was synchronous (electronic supplementary material, figure S6).

### (d) Downstream pleiotropy alters evolved host network structure

While our definition of pleiotropy focused on a shared downstream module or effector (downstream pleiotropy), we allowed for the evolution of additional pleiotropic connections between the signalling proteins of the developmental and immune networks (upstream pleiotropy). In all signalling and resource conditions, hosts with independent effectors were more likely to develop upstream pleiotropic connections than shared effector hosts. This trend was significant in all conditions except when signalling was synchronous and resources were scarce (electronic supplementary material, figure S7). The difference between the two proportions was greater when signalling was asynchronous. Hosts with independent effectors evolved significantly larger signalling networks than shared effector hosts across all resource conditions when signalling was asynchronous. When signalling was synchronous, shared effector and independent effector hosts tended to have similar median network sizes, but shared effector hosts had significantly greater variance in their network sizes than independent effector hosts (electronic supplementary material, figure S8). Hosts with independent effectors were significantly more connected than shared effector hosts across all conditions, with the difference growing more extreme when signalling was asynchronous (electronic supplementary material, figure S9).

## 4. Discussion

Our results suggest that pleiotropy provides a significant competitive advantage to hosts co-evolving with pathogens, providing a fundamental explanation for its maintenance in immune signalling networks across a broad array of taxa despite the potential constraint on trait deployment and evolution [17]. Our results also predict that upstream pleiotropy should be most common in hosts with no downstream pleiotropic module, highlighting a vital role for pleiotropic proteins in inter-network coordination.

We initially hypothesized that pleiotropic effectors would be detrimental to organismal fitness during synchronous signalling and that this detriment would be alleviated during asynchronous signalling because the resource allocation issue between the networks would disappear. The independent evolution simulations partially support our hypothesis; when signalling is synchronous, shared effector hosts have lower average fitness than independent effector hosts under some resource conditions, but this finding is not universal and is even reversed when resources are scarce (figure 1). This limited support is further undercut when considering the results of competitive simulations, where shared effector hosts are highly successful across resource and signalling conditions (figure 2). Interestingly, independent fitness is a poor predictor of competitive fitness, with shared effector hosts winning in more conditions than would be expected based on the independent evolution results (figures 1 and 2). Instead, signalling network robustness and the variance in absolute fitness appear to play a large role in determining which population will win a competitive simulation (figure 3; electronic supplementary material, table S1). Here we have presented evidence that sharing effector modules to coordinate multiple signalling networks can be a highly fit evolutionary end point, particularly when the networks are young, providing a theoretical understanding for the prevalence of pleiotropy in organismal signalling networks.

As expected from well-established theory and empirical studies [16,36,37] we found that resource availability limits absolute fitness (figure 1) and this finding seems to depend on the relationship between resource availability and host signalling network robustness (figure 3). There is significant theoretical backing for the expectation that the resource demands for a system increase as its robustness increases, largely due to the costs of redundancy and the inefficiencies associated with network modularity [38]. We found that both shared and independent effector host robustness increased when resource availability increased (figure 3) and we believe that this increase in robustness can be attributed to an increase in network size as resource availability increases (electronic supplementary material, figure S8). This belief is supported by previous theoretical work which demonstrated that increasing network size is a biologically relevant mechanism to increase robustness [39]. The scarce resource condition would then prevent hosts from assuming robust, but less resource efficient, network configurations (figure 3; electronic supplementary material, figure S8) and this resource derived constraint could then be reduced or avoided entirely by deploying a shared effector to accomplish multiple tasks.

The relative merits of shared and independent effectors depend on the timing of trait deployment. Independent effector host fitness is elevated during asynchronous signalling relative to synchronous signalling, possibly because they can maximize efficiency for both traits; having two effectors that can follow different signalling dynamics improves fitness compared to a single effector that must compromise between optimal dynamics. Shared effector host fitness, on the other hand, seems to be disproportionately elevated during synchronous deployment. In this case, the single effector is capable of simultaneously accomplishing both the developmental and immune task in a resource-conserving manner, minimizing antagonistic competition [40]. If the independent effectors have different costs, or the cost of the single effector balloons for one trait versus another, we speculate that these results would break down. Across resource conditions and hosts, synchronous signalling lowers the variance in host fitness (electronic supplementary material, table S1), which may be associated with reduced population heterogeneity as there are fewer distinct ‘genotypes’ of hosts that compose a population [41,42]. This finding suggests that the synchronous signalling condition requires relatively simple signalling network solutions, leaving less room in the fitness landscape for population heterogeneity.

Shared and independent effector populations evolve significantly different signalling network structures to achieve similar response states (electronic supplementary material, figures S7–S10). Independent effector hosts are more likely to develop upstream pleiotropic connections than shared effector hosts (electronic supplementary material, figure S7), suggesting that hosts with independent effectors must develop inter-network interactions to coordinate effector activity, while shared effector hosts are less reliant on such upstream pleiotropic connections. The coordination of traits accomplished through both up- and downstream pleiotropic connections has a biological basis in resource management; for example, it is vital for arid plant survival in resource-poor environments [43]. Interestingly, upstream pleiotropic connections, which are less connected, do not alter host signalling activity in the same way that downstream pleiotropic connections do (figures 1 and 3; electronic supplementary material, figures S7–S10), reflecting previous observations that pleiotropy, defined as the number of gene–gene interactions, positively correlates with the impact of a gene on host fitness [44].

There is ample evolutionary space in which hosts that share an effector module can thrive, especially when competing against hosts that do not share an effector module. What is less clear, however, is why this phenomenon is occurring. Prior to these experiments we predicted that independent effector hosts should be capable of at least matching, if not exceeding, the fitness of shared effector hosts. This is true during independent evolution, but not during competitive evolution simulations. We believe that the competitive advantage of shared effector hosts in this model is as follows: shared effector hosts generally have a lower variance in population fitness, especially when signalling is synchronous (figure 1; electronic supplementary material, table S1), which imparts a competitive advantage against a more fit but highly variable population [45]. Shared effector host fitness is less variable due to their robustness, as robust immune responses allow them to consistently respond to a range of parasitic manipulations. Shared effector host robustness arises because of their effector module configuration; they possess significantly more connections to their effector than independent effector hosts (electronic supplementary material, figure S10) which positively correlates with robustness (electronic supplementary material, figure S11). Furthermore, shared effector hosts always have lower network connectivity than independent effector hosts that evolved under the same conditions (electronic supplementary material, figure S9), which is also associated with robustness [39,46]. This is important for our competitive simulation because less robust hosts will be more susceptible to large intergenerational decreases in fitness due to parasitic activity. Following such an intergenerational loss of fitness, a population may find it hard to recover against their competitors, especially as a greater proportion of the parasite population begins to target the susceptible population.

A second piece of the competitive puzzle seems to be the rate at which both populations improve their fitness. We observed that the average population fitness of shared effector hosts increases more rapidly than their independent effector counterparts, especially when signalling is synchronous (electronic supplementary material, figure S11), as is expected of pleiotropic adaptation when initial organismal fitness is far away from a theoretical optimum, consistent with theoretical predictions [47] and empirical study [48].

Taken together with the differences between arithmetic and geometric mean fitness, these findings paint a picture of downstream pleiotropy being advantageous, especially in early generations, which is further supported by the increased competitive success that shared effector hosts experience when the populations are allowed fewer generations to evolve prior to competition. The competitive advantage of shared effector hosts is dependent on highly robust signalling networks, rapid adaptability, and the true fitness differences between host populations being less than the arithmetic mean would indicate. Interestingly, the advantage associated with sharing an effector is most pronounced in synchronous signalling conditions (electronic supplementary material, figure S2), contradicting the notion that pleiotropy is detrimental during resource allocation trade-offs and instead providing evidence for pleiotropic effectors being evolutionarily favourable relative to independent effectors.

This work provides a theoretical framework for studying the evolutionary interactions of multiple signalling networks and is especially appropriate for understanding how they share resources and coordinate responses. In turn, these models can help to provide potential evolutionary explanations for seemingly poorly fit behaviours or phenotypes when these phenomena are inaccessible to empirical study. For example, we started this project questioning the role of phenoloxidase as a key component of both cuticle and pathogen melanization when this configuration seemingly leads to resource allocation trade-offs. Our findings propose that in some resource and signalling environments, the current configuration is more fit than using multiple effectors, so the supposed fitness deficit is in fact the most fit implementation of the given biological network. These findings can also help to explain how novel traits rise to prominence in a population: as new traits arise they coopt existing genetic architecture [9,49], and this shared architecture may eventually separate into distinct traits following duplication and subfunctionalization events [44,50]. Our work has shown that the intermediate pleiotropic state, where traits share signalling components, can be well tolerated from a perspective of organismal fitness, and allows for more rapid fitness improvements than would be observed otherwise. We expect these findings to be generalizable to other traits beyond immunity and development, as the major benefit of downstream pleiotropy is an increase in signalling network robustness compared to deploying independent signalling effectors. Signalling network robustness is proposed to be a mechanism to reduce mutational stress on signalling networks [51] and the pursuit of robustness has been shown to drive the evolution of signalling network complexity [39].

Future models could investigate how the stage-structure of trait expression, e.g. in insects with complete or incomplete metamorphosis or in species that experience variable exposure to parasites in different life stages, influences the evolution of pleiotropic signalling networks. In addition, our simulations were carried out assuming that both traits contribute equally to host fitness, but imbalanced contributions may select for entirely different competitive dynamics. Finally, this work focuses on the potential benefits or costs of pleiotropy from a host perspective, but there is substantial evidence that parasites make extensive use of pleiotropic proteins, both for their own purposes [52] and as virulence factors [52,53]. A similar model studying parasites that deploy proteins that have unique functions between species could then be a first step in understanding the evolutionary dynamics that lead to and result from such cross-species functionality. This work provides support for the value of a network-based perspective when studying trait evolution, as we were able to provide novel insights into the evolution of shared genetic architecture with implications both for the evolution and maintenance of genetic pleiotropy as well as network-level resource management strategies.

## Data Availability

The data used to generate the figures in this paper are deposited in Data Dryad: https://doi.org/10.5061/dryad.sqv9s4n9f [54]. The code used to run the simulations and generate the figures can be found within the GitHub repository: https://github.com/Reese-Martin/Pleiotropy_And_Resource_Allocation. Electronic supplementary material is available online [55].
